# Notes and News

**Published:** 1890-08-23

**Authors:** 


### NOTES AND NEWS.

COLLECTED AND COMMUNICATED.

THE Garston Accident Hospital, near Liverpool, is not widely known, but it does good work. It contains five beds, and is under the Local Board of Health; it is only for severe accidents of which there are a great number, owing to the nearness of the London and North-Western Railway docks and some large iron and copper works. During the last year there have been 45 cases admitted, and there were 6 deaths, 3 occurring on admission. This was 20 more cases than the previous year. The honorary medical staff hope before long to have a larger hospital. The matron is Miss E. H. Hunt.

A LARGER number of fever patients are under treatment at the metropolitan hospitals than have ever been known before at this time of the year. The six establishments of the Metropolitan Asylums Board—at Fulham, Stockwell, Winchmore Hill, Homerton, Hampstead, and Deptford—have about 1,649 cases. The excess is due chiefly to scarlet fever, which, however, exists in a very mild form, and latterly has begun to decline. There is reason to hope that there will be no necessity to provide additional accommodation, although as a rule this disease, when epidemic, is most prevalent in September, October, and November.

MR. JAMES NASMYTH, inventor of the steam hammer, leaves a fortune of £243,000. He leaves his residuary estate in trust for division into 100 parts, and he bequeaths four parts thereof to the Hospital for Women, Soho; sixteen parts to the Royal Scottish Academy of Arts, to form a fund for the benefit of decayed artists, to be called the Alexander Nasmyth Fund; and eight parts each to the Artists' Benevolent Fund, the Artists' Benevolent Orphan Fund, the Society for the Prevention of Cruelty to Animals, the Tunbridge Wells Dispensary, the Royal Samaritan Hospital for Women and Children, the Edinburgh Royal Dispensary Convalescent Branch, the Manchester Dispensary, the Edinburgh Asylum for the Blind, Henshaw's Asylum for the Blind, Manchester, and the Workshop for the Blind, Greenwich. Mr. Nasmyth directed that his body should be cremated at the Woking Crematorium, believing that process to be the best way of disposing of the dead and the most beneficial for the health of the living. Mr. Nasmyth died in May last.

THE want of hospital provision in Brixton and the largely populated district further south has stimulated a movement for the erection of a cottage hospital to serve the increasing needs of the local community. In rebuilding its institution the committee of the Brixton Infirmary has provided plans and accommodation in connection with it for the establishment of such an hospital, on the condition that the sum of £2,000 is raised for the purpose. A few workhouse infirmaries are the only places open for the reception of accident patients and other cases of urgency in this extensive neighbourhood, and it is not apprehended that among the wealthy inhabitants of the district there will be much difficulty in obtaining the sum indicated.

THE secretary of the West Bromwich District Hospital writes to correct an error in our issue of August 9th. The following is the correct reading of the resolution to which we referred:—"Your board, in order to carry out the resolution passed at the last annual meeting of governors, have purchased the piece of land in Lodge Road, and have decided to erect thereon two circular wards, to contain 12 beds each, with four small wards adjoining to contain 1 bed each, to remove the mortuary, laundry, &c., to put up a new operating room, to convert the present operating room into a ward with 2 beds, to build additional sleeping rooms for the nurses, and to make sundry minor alterations and improvements." The cost of these new buildings will be about £6,000.

A CORRESPONDENCE has been going on in the *Morning Post* on the question of the titles, or rank, which army surgeons should hold. The proposal that they should apply for the title of Colonel we are glad to see has not evoked applause. A "Military Doctor" writes and points out that the naval surgeons are content, and hence draws the deduction that the present evil in the army in not the lack of title, but the lack of attachment to a special regiment: "We, on the other hand, by a system that removed us from regiments, are isolated from the rest of the army; are knocked about from pillar to post; do not know our patients, nor are we known of them; and, in fact, feel as 'Hauptmann' (rather unkindly) calls us, civilians. Hence the cry of some for military titles, in order that they and their friends may be able to grasp their connection with the army, while others, like myself, still prefer being "doctor," and waiting wearily for the time when, by a return once more to the old system, they will be able to feel that while without encroaching on the rights of the combatant branch, their position as a regimental officer will be ensured."

THE matron of the Maghull Home for Epileptics has very kindly sent us a first annual report of that institution; also a medical report which will be dealt with later on. The home was established chiefly through the efforts of the late Mr. Henry Cox and Dr. William Alexander. The year began with a solitary patient, and ends with twenty-two in the home, and several females anxiously awaiting a vacancy. Thirty-one patients have been admitted up to the end of the year and nine discharged. Five were second class, and twenty-six third class. The female side of the home has been long filled up; there are vacancies for two third class males, and for several more first or second class patients. There is no doubt that the question of more room will soon become a pressing one; for females, indeed, it is so already. Miss Paley, matron, is a trained nurse, and is assisted by a qualified nurse and a probationer. The patients have to pay according to their means, though as soon as the committee can afford it, they hope to take a few free patients. The chief point seems to be that the "home" is not a hospital; the patients are expected to work and to behave as in an ordinary house; they are kept occupied and amused, and are sensibly fed. The result is very marked improvement in one-third of the cases, while all improved physically.

The following vacancies are announced this week, the amount of the salary in each case being given after the name of the institution:—

**Resident Medical Officers.**

Taunton and Somerset Hospital, House Surgeon—£100, board, etc.

Wrexham Infirmary and Dispensary, House Surgeon—£80, board, etc.

Dewsbury Infirmary, House Surgeon—£80, board, etc.

Burntwood Lunatic Asylum, Assistant Medical Officer—£120, board, etc.

Brompton Consumption Hospital, Resident Medical Officer—£200, board, etc.

Ryde Infirmary, House Surgeon and Secretary—£60, board, etc.

Manchester Royal Infirmary, Resident Surgical Officer—£150, board, etc.

Manchester Children's Hospital, Medical Officer—£180, board, etc.

Ramsgate Royal Dispensary, Medical Officer—£120, rooms, etc.

Birmingham General Hospital, Resident Surgical Officer—£130, board, etc.

Jessop Hospital for Women, House Surgeon—£50, board, etc.

British Hospital, Buenos Ayres, Resident Medical Officer—£200, board, etc.

Walsall Friendly Societies, Medical Officer—£200, rooms, etc.

**Non-Resident Medical Officers.**

County Council of Ross and Cromarty, Medical Officer of Health—£300.

County of Argyll, Medical Officer of Health—£350.

County of Renfrewshire, Medical Officer of Health—£500.

Manchester Consumption Hospital—Hon. Assistant Physician.

---

The District Hospital of Billericary has been burnt down. Luckily there were no patients in at the time, but the resident nurse, Mrs. Lacey, was rescued with some difficulty. The buildings were almost wholly of wood, and though the fire brigade was promptly in attendance, the flames spread with such rapidity that everything was utterly destroyed in an hour and a-half.

---

The Duchess of Albany last week presented certificates to the successful pupils of the Hammersmith branch of the St. John's Ambulance Association. The hon. secretary, Mr. T. Freeman, read an address of gratitude to her Royal Highness for the interest she had shown in the centre, and mentioning after allusion to the valuable services of teaching rendered by Dr. Forbes Winslow, Dr. Fountain, and Drs. Barnes, Dodsworth, Mahon, and Walker, that out of the one hundred and one candidates who had presented themselves for examination by Dr. Matthew Coates, no fewer than ninety-three had passed, some with very great distinction. The Duchess then distributed the awards, many of which were taken by the police from the district, who have attended the classes with great regularity and interest. A short practical demonstration of "First Aid" work was then given, and General Goldsworthy proposed a vote of thanks to the Princess for her kindly presence.

---

The annual report of Horton Infirmary has just reached us. The tabular statement of the house surgeon shows that the number of cases treated in the infirmary during the past year was 328, whilst 360 accidents and urgent cases have been relieved, but not taken in as resident patients, making a total of 788 medical and surgical cases, to which must be added the dental cases reaching to the large number of 395, making a total of 1,183 persons, whom we trust have been in a greater or lesser degree benefitted by the institution. The average number of beds occupied were 27, as against 25 in the previous year, whilst the largest at any one time was 31 in February, 1890. The books of the provident dispensary for the year ending December 31st give 1,073 patients, and receipts amounting to £171 9s. 7d., showing that the institution is doing good work amongst our industrious neighbours. The house surgeon and the matron are thanked for their services. Every report which we have to read increases our desire for a uniformity in charity accounts. No two reports are made up in the same manner, and to compare them is, therefore, ever a work of time.

In his weekly-published report, Dr. Tatham, Medical Officer of Health for the City of Manchester, says that although each returning autumn furnishes confirmatory evidence of the fact that children taken to the seaside for the benefit of their health frequently contract scarlet and other infectious fevers at these "health resorts," it is not a little astonishing that heads of families continue to manifest so little solicitude that the quarters they select shall present a clean bill of health. As the watering places on our coast mainly depend for their support on visitors from inland towns, the latter are entitled to a guarantee that the seeds of infectious disease and death shall not be actively present in apartments offered for their occupation. It is fortunate that at the most popular of our health resorts powers are already in operation for the compulsory notification of infectious disease, and visitors who are anxious for the safety of their families may accordingly obtain reliable information from the local medical officer of health. It is not generally known that Table 8 of the Registrar General's quarterly report gives the deaths from infectious diseases at the different watering places—a record which may be studied with advantage by those desiring a holiday resort for their family.

---

Miss Twining says it may interest our readers to know that in the June number of the *State Charities Record*, published in New York, there is an interesting article, "A Plea for the Epileptics," by Dr. Peterson, of that city. He states what is probably unknown to many, that much has already been done for this class of sufferers in Germany (the institutions of M. Bost, in France, are better known, having been many years established). Dr. Peterson wrote a description of the Bielefeld Colony in an article on "The Colonization of Epileptics," in the "Journal of Nervous and Mental Diseases," December, 1889. This institution was begun 24 years ago near Hanover, and, in 1887, it consisted of 55 houses and 320 acres of land, with schools and workshops for 30 different trades and callings, all the work of the village being done by the patients, both men and women. Nine other such colonies have followed this model in Germany, besides one in Holland and one in Switzerland. The usual objection to collecting vast numbers of one particular class of disease together does not apply here, as I believe it is known and acknowledged as a fact, that epileptics do not injure or affect each other by contact, while they do great mischief to those, especially the young, who are not similarly afflicted. There is nothing more done in America for this class than in England.

---

The *Scotsman* for August 9th contained a brief summary of the medical charities of Edinburgh, which was of considerable interest. St. Kessop's Home, the Homœopathic Dispensary, and other unfamiliar charities figured in conjunction with the Royal Infirmary and Sick Children's Hospital. With regard to the dispensaries and their needless multiplication, it was stated that in the case of the two dispensaries which are nearest to one another, the Royal Dispensary and the Provident Dispensary, the supply in the district seems slightly greater than the demand. The result is that the older institution finds itself being gradually superseded in public favour by its younger and more active rival. The number of persons treated by the Royal Dispensary has been gradually falling off during the last three years, while during the same period the number given by the Provident Dispensary shows an increase. In 1887 they treated 8,084 persons; in 1888, 8,216; and last year they ran up to 9,957. Popular favour is the best guide by which to determine the efficiency of such an institution, and it seems to have decided for the Provident. The management of the Royal Dispensary have recognised this, and have rather unwarrantably devoted a portion of their funds to the erection of a School of Pharmacy, a venture hardly contemplated when the Dispensary was endowed.

